# Association Between Tooth Loss and Bone Mineral Density in Brazilian Postmenopausal Women

**DOI:** 10.4021/jocmr513w

**Published:** 2011-05-19

**Authors:** Paulo Sergio Gomes Henriques, Aarao Mendes Pinto Neto

**Affiliations:** a Department of Periodontics, Faculty of Dentistry, Sao Leopoldo Mandic, Campinas, Sao Paulo, SP, Brazil; b Department of Obstetrics and Gynecology, CAISM, Faculty of Medical Sciences, Universidade Estadual de Campinas, Sao Paulo, SP, Brazil

## Abstract

**Background:**

To evaluate oral health in postmenopausal women and verify whether there is a correlation between tooth loss according to index of decayed, missing, filled teeth (DMFT) and bone mineral density (BMD).

**Methods:**

A cross-sectional study was conducted with 100 women. The DMFT and its associations with lumbar and femoral BMD (T-score and g/cm^2^) were assessed. Analysis of covariance and multiple logistic regression were applied and the mean and standard deviation, absolute and relative frequencies (percentages) were obtained.

**Results:**

The analysis of covariance (ANCOVA) revealed significant association between the DMFT index and bone mass (T-score), compared to the young adult in L2-L4 (P = 0.0252) and in bone mass in L2-L4 (below average) in g/cm^2^ and in the DMFT index (P = 0.0332), and for women with bone mass below the average index DMFT was higher. Between L2-L4 below average (g/cm^2^) and extracted component (P = 0.0483) association was also significant because women with bone mass below the average had a greater extracted component.

**Conclusions:**

Postmenopausal women with poor oral health may present reduced bone mass. There was significant association between BMD and DMFT at the L2-L4 site. Women must be advised that their good oral health, amount and quality of bone mass should also be matters of concern.

**Keywords:**

Bone mineral density; Post-menopausal; Osteoporosis; Tooth loss

## Introduction

Brazil has over the last few decades seen a raise in life expectancy, now at 73 years of age. This will make, in 2025, the country with the sixth oldest population in the world. As a result, there will be more age related diseases [1].

The menopause is a physiological event in which there is an interruption in the menstrual cycle and results in hormonal changes that are irreversible. Around the age of 50, some signals can be seen such as flushes, sudorose, depression, irritability, loss of concentration, dry skin, and osteoporosis [2].

Osteoporosis is the most common metabolic bone disease in the world. Due to the increase in this disease, it has been questioned as to whether this disease has any relation to the loss of teeth [3, 4].

There is an estimate that osteoporosis affects 75 million people in the USA, Europe and Japan, including 1 in 3 women in the post menopause stage, most of which are elderly. Today there are 10 million Americans with osteoporosis and 18 million with loss of bone mass. The cost that comes, including emergency calls, surgical treatment, physiotherapy and rehabilitation, and time missed from work, just in the USA, is approximately 20 billion dollars annually. This would be enough to accelerate preventive public programs with strategies aimed at reduction of social and economic costs [3-6].

Densitometry is the most common technique used to test the bone mineral density. It is important to make an early diagnosis of bone loss before the occurring of osteoporosis [7].

Studies have shown the correlation between the bone mineral density and tooth loss [8-11]. Women in the postmenopausal stage, with normal bone mineral density, loose an average of 6.8 teeth, while women with osteopenia loose 10.5 teeth and with osteoporosis loose 16.5 teeth [12].

There is a reported correlation between tooth loss and high prevalence of fracture in the lumbar spine [13]. There was also a positive relation between the loss of posterior teeth and the bone mineral density in the spinal column. Studies suggest that women with a high bone mineral density tend to have more teeth than those with low bone mineral values [14].

The estrogenic deficiency after the menopause is considered to be a possible cause that relates the loss of teeth to the low bone mineral density in women. The long-term use of hormonal therapy (10 years or more) shows an average of the decrease in tooth loss by 3 teeth, compared with those who do not use the therapy [15-17].

Cavities and periodontal diseases cause the tooth loss [18-20]. The alveolar processes in the upper and lower jaw provide the bone structure that provides the support for the teeth. The osteoporotic changes in these bones could directly affect the retention and stability of the teeth. This suggests that the severity of osteopenia and osteoporosis is related to loss of height of the alveolar crest and tooth loss in women after menopause [21, 22].

Genetic factors, in particular, certain polymorphisms of the gene that regulates interleukin-1, are an important mediator of inflammation and bone destruction in the periodontium. The gene for vitamin D receptor has been identified, with possible polymorphism influencing the low bone mineral density, and some other studies link it with the dental loss [23].

Strategies for training and prevention of osteoporosis may bring additional benefits in the reduction of tooth loss [24].

This study intends to observe the association between the DMFT index and its individual components with bone mineral density at the lumbar spine and femoral neck in Brazilian women.

## Materials and Methods

The study included one hundred women in post-menopausal stage at the Menopause Outpatient Facility of the Center of Integral Attention to Women’s Health (CAISM) at the State University in Campinas, who had undergone densitometry test in three years preceding the evaluation and were amenorrhea for at least 12 months. The women who accepted and met the inclusion criteria and participated in the study were ensured confidentiality of information and the patients were identified with numbers.

The principles enunciated in the Declaration of Helsinki amended in Scotland (2000) were complied with and the study was approved following the guidelines and regulations for research involving human subjects.

Data collection was performed during a routine consultation of the women in the menopause clinic of probable causes and the medical record containing the bone densitometry held in the Nuclear Medicine Department (all women had the same type of test/densitometry). The volunteers were informed about the study and signed a consent form and answered a questionnaire that addressed the age in complete years, age of menopause and the time of the last menstruation, menopause time in years between the last day of menstruation and evaluation, use of antireabsorptive agents, hormone replacement and/or bisphosphonates, smokers, and number of years of study.

Then a full examination of the oral cavity was carried out by a single evaluator who underwent training for use of the criteria for diagnosis of caries that was used. We used the DMFT index that measures the amount of permanent teeth decayed, missing, filled in the individuals mouth, ranging from 0 to 32 [25].

The densitometry measured BMD at various skeletal sites, especially the lumbar spine (L2-L4 segment) and femur (neck and trochanter) [26].

Bone mineral density and bone mass measurements were categorized as values for the T-score (young adult) that corresponds to the number of standard deviations above or below the average young adult of reference, classified as normal up to -1 SD, osteopenia from -1.0 to -2.5 SD and osteoporosis below -2.5 SD, and as absolute values in g/cm^2^ which measured bone mass in g/cm^2^ and were expressed through being above and below the mean and standard deviation 1.

The data was initially evaluated in a descriptive way, with calculation of means, standard deviation and absolute and relative frequencies and percentages, in the case of continuous variables. The Student t test to compare the mean DMFT index in women with and without osteoporosis, and analysis of covariance (ANCOVA) to study the association between the DMFT index and BMD were used controlling for covariates, age, age at menopause, use of antireabsorptive drugs, smoking and education. The level of significance was set at 5% (P < 0.05).

## Results

**Table 1:** Relation Between DMFT Index and Bone Mass (T-score) in L2-L4 and Femoral Neck (Covariance Analysis)

Variable	Estimated parameter	P*
DMFT index		
L2-L4**	3.5929	0.0252
Femoral neck**	1.9311	0.3235
Trochanter**	0.035	0.8256
Decayed teeth		
L2-L4**	0.2635	0.2278
Femoral neck**	0.2126	0.4307
Trochanter**	0.0398	0.8749
Filled teeth		
L2-L4**	0.9982	0.3766
Femoral neck**	0.9287	0.5061
Trochanter**	2.1886	0.0985
Missing teeth		
L2-L4**	13.5239	0.0719
Femoral neck**	8.2726	0.3696
Trochanter**	5.4540	0.5285

* Control variables: age, age at menopause, use of antireabsorptive drugs, pack years of smoking and education level. ** values in changed T-score (osteopenia or osteoporosis).

The analysis of covariance (ANCOVA) revealed significant association between the DMFT index and bone mass (T-score) compared to the young adult in L2-L4 (P = 0.0252), and for women with altered values (osteoporosis) the DMFT index was the highest (estimated parameter > 0) (Table 1).

**Table 2:** Relation Between DMFT Index and Bone Mass (g/cm^2^) in L2-L4 and Femoral Neck (Covariance Analysis)

Variable	Estimated parameter	P*
DMFT index		
L2-L4**	3.3545	0.0332
Femoral neck**	-1.6079	0.3986
Trochanter**	2.9615	0.1187
Decayed teeth		
L2-L4**	-0.2129	0.3249
Femoral neck**	0.1295	0.6248
Trochanter**	-0.1494	0.5688
Filled teeth		
L2-L4**	1.5427	0.1757
Femoral neck**	0.4381	0.7526
Trochanter**	0.0934	0.9458
Missing teeth		
L2-L4**	14.6387	0.0483
Femoral neck**	6.7986	0.4492
Trochanter**	9.4297	0.2900

* Control variables: age, age at menopause, use of antireabsorptive drugs, pack years of smoking and education level. ** Estimated bone mass values below average.

The analysis of covariance (ANCOVA) revealed significant association between bone mass in L2-L4 (below average) in g/cm^2^ and DMFT index (P = 0.0332), and for women with bone mass below the average index DMFT was higher. Association between bone mass in L2-L4 (below average, g/cm^2^) and extracted component (P = 0.0483) was also significant because women with bone mass below the average had a greater extracted component. Women with BMD change had higher DMFT index and major extracted component (Table 2).

## Discussion

The main finding of this study indicates that there were significant associations between the DMFT index and bone mass change (T-score) when measured in L2-L4, between DMFT index and extracted component and the bone mass below-average (g/cm^2^).

The women studied had a mean age of 57.4 ± 8.3 years. The literature has shown that individuals with more than 60 years have poor oral conditions in relation to the index DMFT [27, 28]. The clinical investigation showed an increasing amount of tooth loss, root caries, and periodontal disease with increasing age [29].

In this study, most women reported limited schooling, because only 10% of them had high school level or higher. This corroborates with the literature that shows the association between low education level and osteoporosis and increased tooth loss [11].

More than 70% of the women studied were non-smokers, despite the longitudinal studies showing that pack years of smoking is a risk factor for decreased bone density in women in post-menopause. Smokers also exhibit greater tendency to progressive loss of teeth [17, 30, 31].

It is important to note that more than half of women in the study were users of hormonal therapy. In women after menopause the relative risk of tooth loss increases 1% each year, with the systemic decrease in the bone mass. Every 4.2 years of use of estrogen, we observed the average retention of one tooth. Moreover, the prolonged estrogen use (> 8 years), keeps on average 3.6 teeth [32].

A study reporting hormone replacement after menopause and dental retention, found that users of estrogen had more teeth than non-users (12.5 × 10.7; P = 0.046). The estrogen was shown to be a protector of tooth loss and bone loss after menopause [33] and estrogen deficiency influenced the reduction in BMD [34].

It was observed that women with altered BMD showed a higher index of DMF, and the extracted component. Studies suggest that a small number of remaining teeth may be associated with low BMD in women after menopause, due to insufficient intake of calcium [14]. Estrogen deficiency at this stage also has been reported as an important cause [35].

We found high average DMFT index (26.7 ± 5.9), and the extracted component (71.1 ± 30.2%), results similar to those of the “Epidemiological Survey on Oral Health” held in Brazil, which showed a broad review in the age group of 50 to 59 years, where the DMFT index was 27.2 and with 86% share of the extracted component [36].

Several authors have linked tooth loss to bone skeletal mineral density in women in the post-menopausal stage [14-16, 37]. However, not all have shown this relation between tooth loss and bone mineral density [38-40].

The analysis of the matrixes performed in this study found that women with altered bone mass in lumbar spine had higher DMFT index, which was adjusted for age, age at menopause, use of antireabsorptive drugs, smoking and education.

The association between the number of remaining teeth and the bone mineral density of lumbar spine and femoral neck was investigated and showed a significant association in the femoral neck, but not in the lumbar spine. Retention of four teeth was significantly associated with an additional gain of 0.004 g/cm^2^ the femoral neck (P < 0.01) [35].

In one study, women with osteoporosis had lost 6.9 teeth, compared to loss of 4.5 teeth in women with BMD normal (P < 0.05) [41]. Another study found that 20% of individuals with osteoporosis were edentulous, while only 7% of those with normal BMD had edentulous [42]. A study of 329 women in post-menopause showed a significant relationship between the number of missing teeth and normal BMD (P < 0.05) and radius (P < 0.01) [16]. However, little is known about the association of teeth and caries and BMD, even knowing that caries can be a cause of tooth loss [43]. In this study it was not possible to know if the tooth loss presented was related to caries or periodontal disease despite the mean number of missing teeth was 22.8 in this population.

The supplementation with calcium and vitamin D has been studied, showing significant reduction of tooth loss in the elderly in a period of three years. There was also an increase in bone mineral density and decreased risk of tooth loss [20, 44].

The better relationship between dentists and physicians, proposing changes in lifestyle of patients with joint actions for prevention, evaluation and treatment of oral diseases and osteoporosis in pre and post-menopause, can bring additional benefits in the reduction of tooth loss and the negative effects of decreased bone mass, this would yield a longer life with better oral health for women.

In conclusion, the findings of this research show that women in post-menopausal stage with low BMD have higher tooth loss and that these losses may also be related to low education and poor oral hygiene, all the concepts related to social vulnerabilities and inadequate oral health programs offered to the population.

To further our understanding of the relationship between skeletal health and oral health, longitudinal studies are needed.
